# Visual bibliometric analysis of cytokines in the pathogenesis of cerebral palsy

**DOI:** 10.1097/MD.0000000000046283

**Published:** 2026-01-16

**Authors:** Zhiliang Cao, Yan Yang

**Affiliations:** a The First Hospital of Hunan University of Chinese Medicine, Changsha, Hunan Province, China.

**Keywords:** bibliometric science, cerebral palsy, cytokines, knowledge map, pathogenesis, visual analysis

## Abstract

**Background:**

Cerebral palsy (CP) is the most common movement disorder in childhood, and its pathogenesis is complex, and cytokines have been shown to be involved in the pathological process of CP as key mediators, but the existing studies have been scattered and lack systematic summary.

**Objective:**

Reveal the research hotspots and cutting-edge trends of cytokines in the pathogenesis of CP.

**Methods:**

A systematic search was conducted in the Web of Science core database for relevant literature on the role of cytokines in the pathogenesis of CP. The CiteSpace and VOSviewer software were used to draw visual maps of authors, institutions, countries, and keywords.

**Results:**

A total of 39 countries, 420 institutions and 1323 authors were identified. The United States led in the number of published articles and was the core of international cooperation. However, the cooperation among authors and institutions was still relatively loose. A total of 1527 keywords were involved. The high-frequency keywords include “inflammation,” “periventricular leukomalacia,” “necrosis-factor-alpha,” etc. Cluster analysis divided the research into 4 categories: inflammation and immune response, perinatal injury, genetic regulation, and other potential associated factors. Emergent analysis shows that the research has gradually delved from basic factors such as early infection and birth weight to cytokine mechanisms and recent studies on inflammation and the child population.

**Conclusions:**

The research on cytokines in the pathogenesis of CP focuses on core areas such as inflammation and immune response, perinatal injury, etc. The research has gradually delved from basic factors to mechanisms and inflammation studies in the pediatric population. In the future, we can pay attention to the dynamic evolution and development trend of these hot areas, optimize research directions and methods in a timely manner, and actively build a multi-dimensional and integrated research system related to cytokines and CP, so as to provide new ideas and strategies for early prevention, targeted therapy and precision rehabilitation of CP.

## 1. Introduction

Cerebral palsy (CP) is a disorder characterized by abnormal posture control, muscle tone and movement, often accompanied by cognitive, sensory, perceptual, communication and behavioral disorders, as well as epilepsy and secondary muscle and bone problems, caused by nonprogressive brain damage in the developing fetus or infant.^[1]^ Epidemiological studies show that 2 to 3 out of every 1000 live births have CP, with a prevalence rate of 2‰ to 3‰.^[2]^ Although brain injury and maldevelopment are recognized as direct causes, risk factors such as maternal infection, prematurity, and perinatal insults contribute significantly to its etiology.^[3,4]^ The exact pathogenesis remains unclear, hampering the development of targeted therapies. Growing evidence implicates immune-inflammatory mechanisms, particularly cytokines, in CP pathogenesis. These signaling molecules mediate intercellular communication during immune responses, and aberrant cytokine levels may contribute to neural damage and neuroinflammation, potentially serving as key intermediaries in CP-related injury.^[5,6]^ However, the diversity and complexity of cytokines, combined with fragmented research, complicate systematic understanding. CiteSpace and VOSviewer are powerful bibliometric software that can efficiently process and analyze large-scale scientific literature data by applying bibliometric methods and visualization analysis techniques, revealing the laws and trends of discipline development.^[7]^ This study integrates these approaches to identify core themes and emerging directions in cytokine-related CP research, offering a holistic perspective on the field and supporting future mechanistic and therapeutic investigations.

## 2. Data and methods

### 2.1. Data sources

Web of Science (WOS) is one of the world’s most influential citation databases, adhering to strict journal selection criteria and featuring over 21,000 high-quality peer-reviewed journals from around the world, covering multiple subject areas, with high academic authority and international representation. The database provides highly structured literature data with complete and standardized fields such as authors, institutions, references, and keywords. CiteSpace software is highly compatible with WOS data formats and can effectively support a variety of bibliometric analyses such as cooperative networks, co-occurrence analysis, and cluster inference, ensuring the accuracy of the research process and the reproducibility of the results. Therefore, WOS was chosen as the core literature source for this study.

### 2.2. Literature search

The literature was sourced from the WOS core database and advanced retrieval was adopted. The search terms were “cerebral palsy,” “brain paralysis,” “cytokine,” “pathogenesis,” etc. The search formula is set as (TS = (cytokine* OR interleukin* OR TNF* OR IFN* OR chemokine* OR TGF-?) OR “tumor necrosis factor” OR “interferon” OR “chemokine” OR “transforming growth factor”)) AND (TS=(“cerebral palsy” OR “spastic diplegia” OR “brain palsy”)) AND (TS = (pathogenesis OR mechanism* OR etiology OR “molecular mechanism” OR inflammatory mechanism OR pathophysiology OR pathway. The search period is set from the establishment of the database to June 5, 2025. The source is limited to journal articles and the language is English. After retrieval, a total of 495 literatures were obtained.

### 2.3. Screening criteria

*Inclusion criteria*: The research topic involves the role of cytokines in the pathogenesis of CP; The type of literature was clinical research, systematic review or high-quality review. *Exclusion criteria*: Conference abstracts, editorials, case reports, letters, and other non-research literature; Incomplete literature (such as lack of authors, affiliations, keywords, or the full text cannot be obtained); Repeatedly published literature.

### 2.4. Data screening

**Figure 1.**
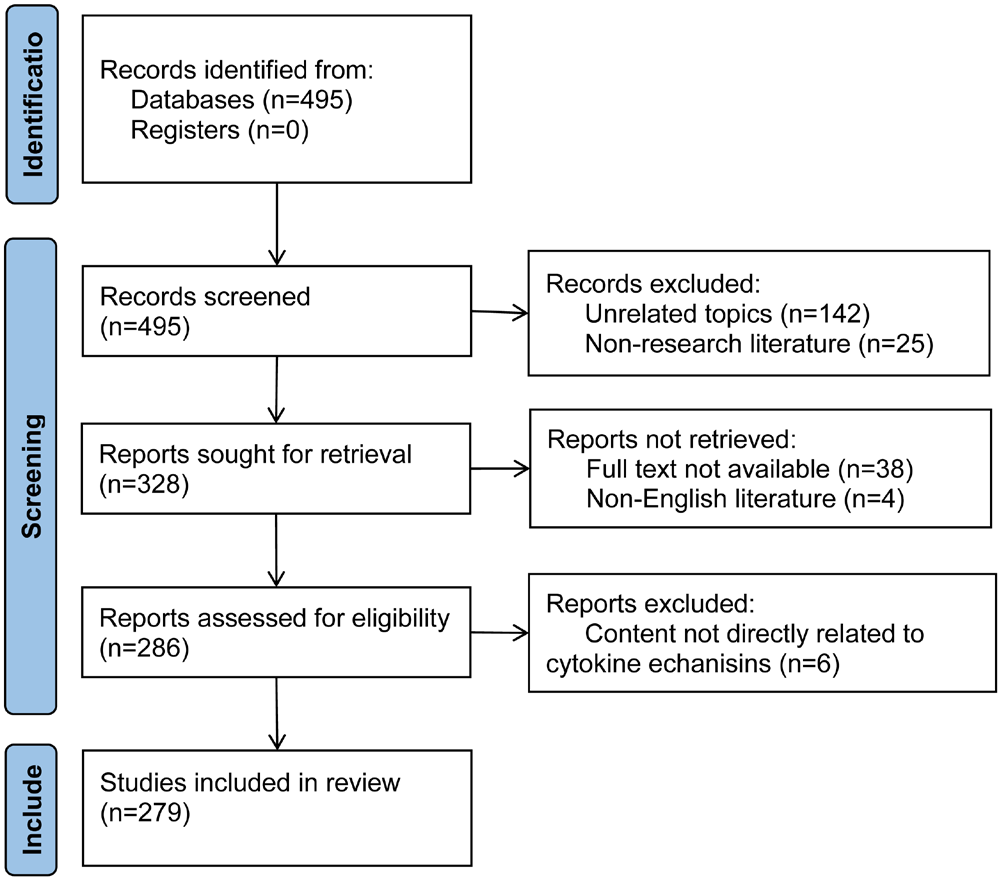
Literature screening procedure.

After the data was exported, it is entered into the NoteExpress 3.2 (Xi’an: Aegean Software Company; 2023) for screening, and duplicate literatures are removed. Two researchers screened the literature one by one according to the screening criteria by reading the titles, keywords and abstracts. When they were unable to determine, they read the full text. In case of differences, they negotiated with a third party. After screening, 279 literatures were finally included. The specific screening process is shown in Figure 1.

### 2.5. Visual analytics

The included literature was exported in the “Plain text file” format in WOS. Citespace 6.3.R1, VOSviewer 1.6.20 were used, and visual analysis was conducted in combination with Scimago Graphica and Pajek V2.0. Figure 1 shows the visual analysis strategy. VOSviewer was used to build author collaboration networks and keyword co-occurrence graphs. CiteSpace was used for keyword clustering, emergence analysis, and timeline mapping. Before analysis, the time span was set to 1996–2025, the time slice was set to 1 year, the node filtering threshold was set to the first 50 per slice (Top N = 50), and the trimming methods were Pathfinder and Pruning sliced networks. The log-likelihood ratio algorithm was used for keyword clustering, and the module value (*Q* > 0.3) and the mean profile value (*S* > 0.7) were used as the structural significance and clustering credibility indicators, respectively. The determination of core authors is based on Price’s law, and authors with no <3 articles are identified as core authors.

## 3. Results

### 3.1. Trend analysis of publication volume

**Figure 2.**
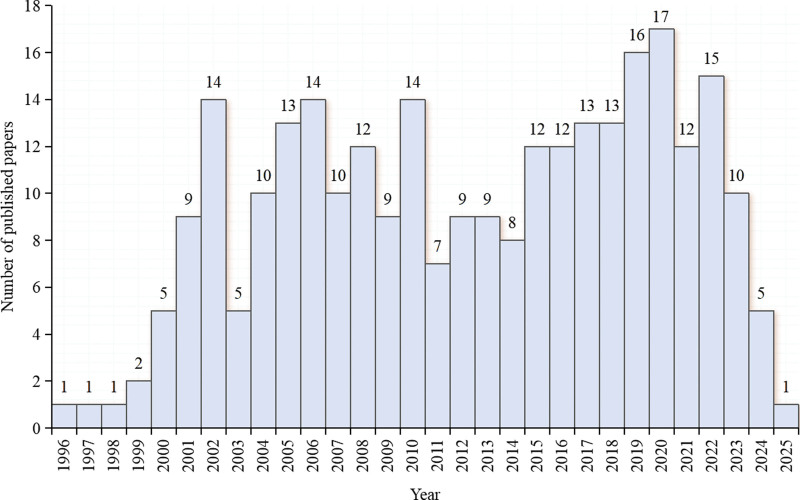
Trend chart of the number of published articles.

From 1996 to 2025, the number of published papers on cytokines in the field of CP pathogenesis showed an overall fluctuating trend. The research enthusiasm was relatively stable, with an average annual number of published papers of 9.3 and a peak of 17, as shown in Figure 2.

### 3.2. Research on national visualization analysis

**Figure 3.**
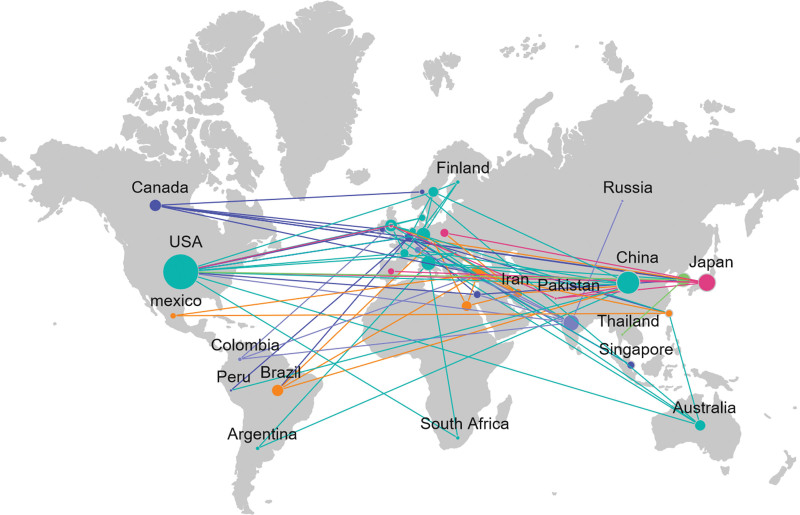
World map of the document issuance situation of each country/region. The larger the node, the higher the number of published articles. The global cooperation network is divided into 6 clusters. Points with the same color are in the same cluster, and connections represent the establishment of cooperation.

**Figure 4.**
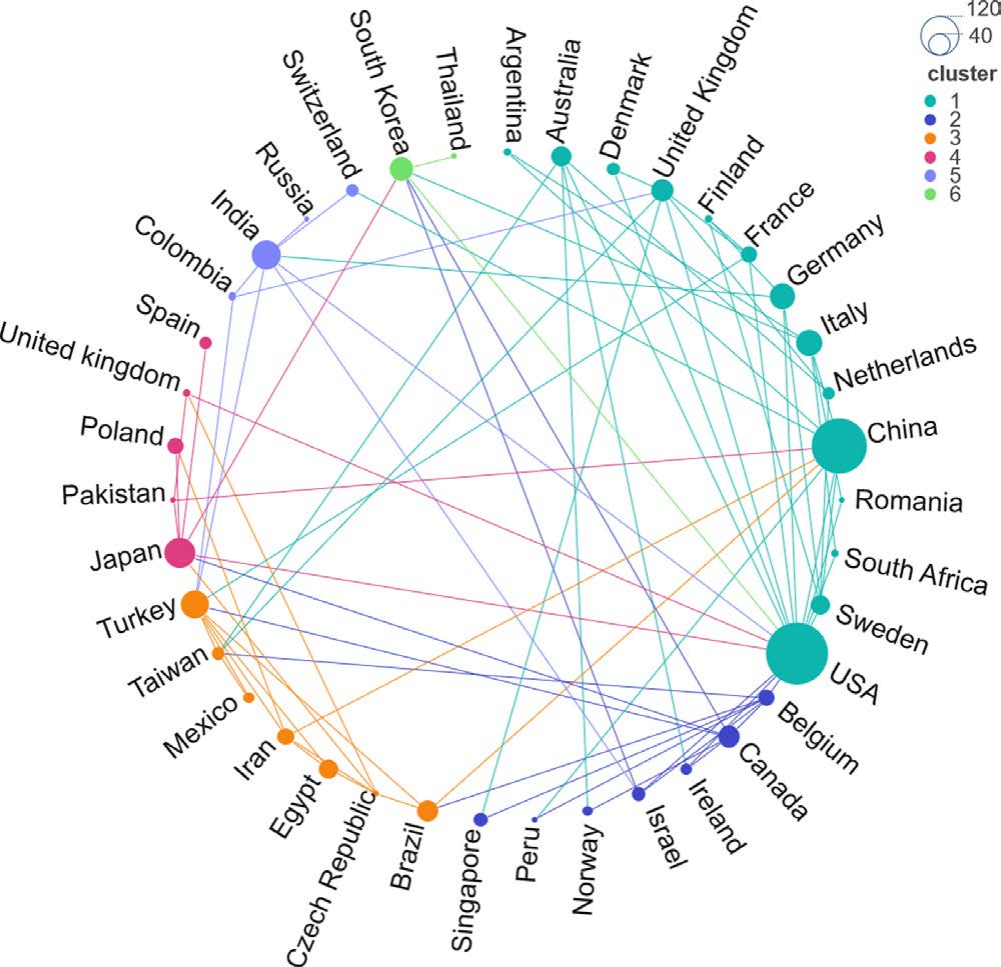
International cooperation network. The larger the node, the higher the number of published articles. The global cooperation network is divided into 6 clusters. Points with the same color are in the same cluster, and connections represent the establishment of cooperation.

A total of 39 countries were involved. The United States had the highest number of published articles (136), as shown in Table 1. The network map shows that the United States was in a central position, closely connected with many countries, and has significant influence, in addition, countries with high scientific research output such as China, Germany and the United Kingdom may become key cooperation nodes, as shown in Figures 3 and 4.

**Table 1:** The top 10 countries in terms of the number of published articles.

Country	Volume of publications	Total link strength
USA	136	52
China	31	13
Australia	21	11
United Kingdom	20	24
Sweden	19	28
France	18	23
Canada	18	8
Germany	13	17
Belgium	13	14
South Korea	11	11

### 3.3. Visualization analysis of institutional cooperation network

**Figure 5.**
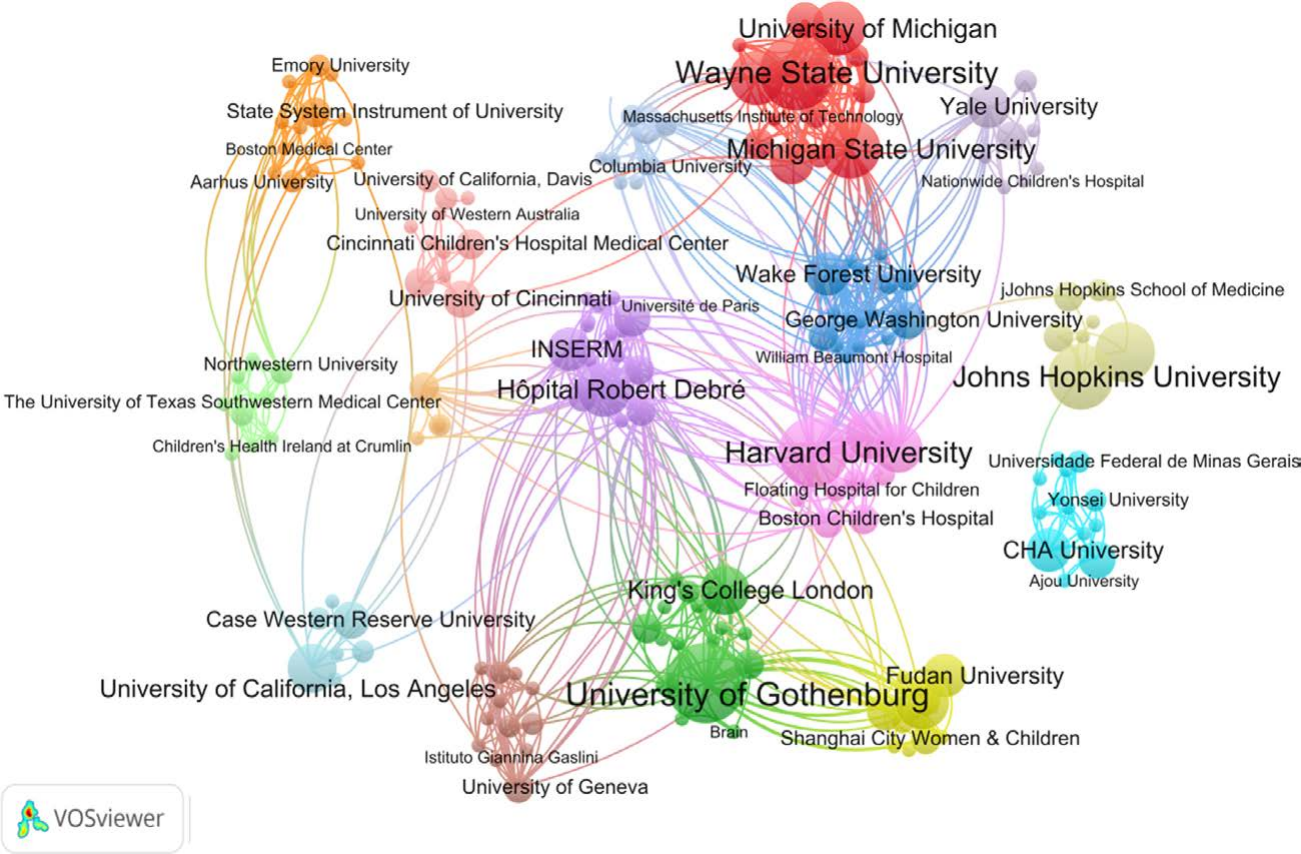
Institutional cooperation co-occurrence map. The node is the name of the institution. Its size is positively correlated with the number of published articles, and the connection line indicates the cooperative relationship.

A total of 420 institutions were involved. The one with the highest number of published papers was Gothenburg University (12 papers), as shown in Table 2. According to the institutional co-occurrence network graph, 16 major cooperative teams centered on Wayne State University, University of Gothenburg, Johns Hopkins University, etc, covering 187 institutions, generated 692 collaborations, and a total chain strength of 812, as shown in Figure 5. The institutions had not yet formed a close and extensive academic cooperation network.

**Table 2:** The top 10 institutions in terms of the number of published articles.

Organization	Volume of publications	Total link strength
University of Gothenburg	12	45
Wayne State University	11	40
Harvard University	10	39
Monash University	9	16
Johns Hopkins University	9	8
Michigan State University	8	55
Université de Sherbrooke	8	9
University of Pennsylvania	8	3
Eunice Kennedy Shriver National Institute of Child Health and Human Development	7	41
Michigan State University	7	36

### 3.4. Visualization analysis of author collaboration networks

**Figure 6.**
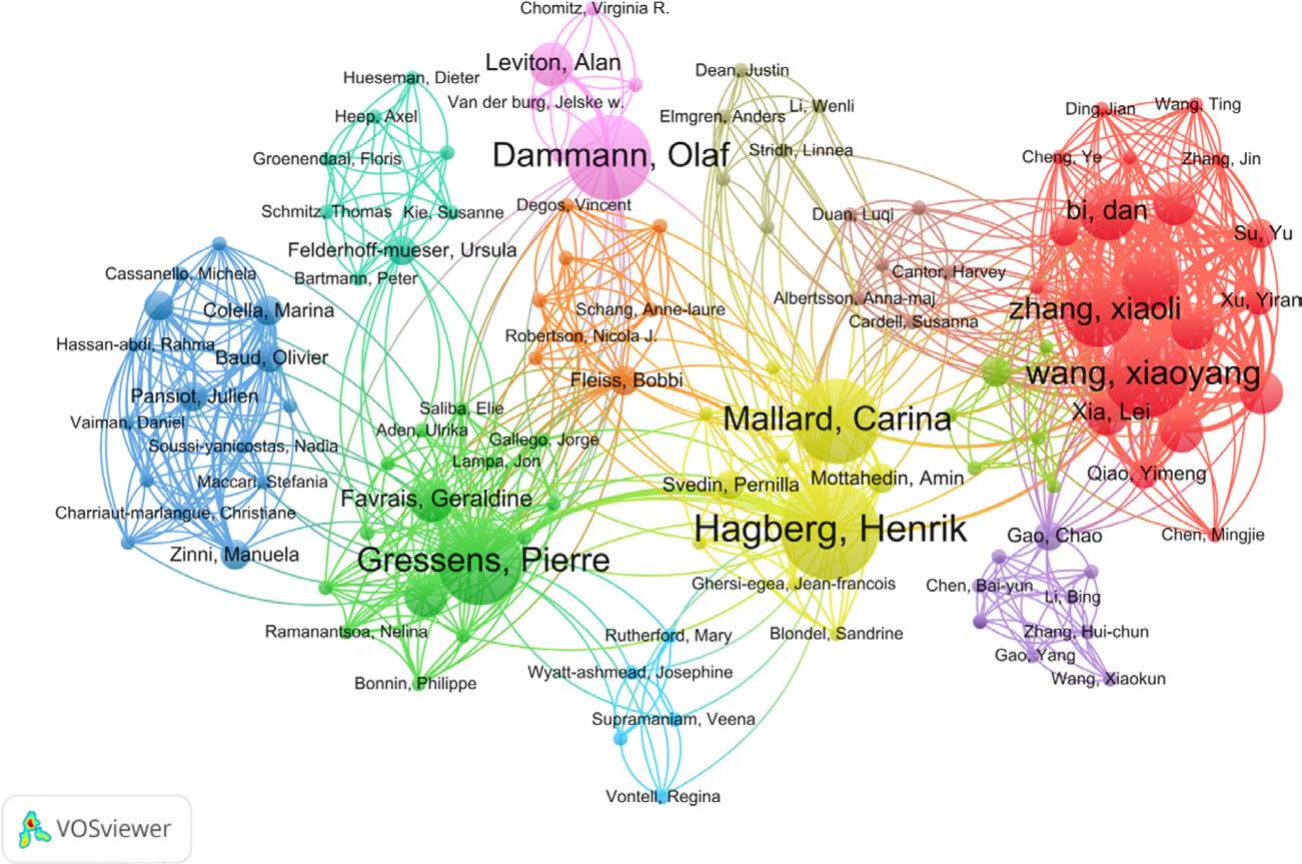
Author collaboration co-occurrence map. The node represents the author’s name. Its size is positively correlated with the number of published articles, and the connection line indicates a cooperative relationship.

A total of 1323 authors were involved. The one with the highest number of published articles was Romero roberto (10 articles), as shown in Table 3. According to Price’s Law, authors with 3 or more starting articles were regarded as core authors, totaling 57, accounting for only 4.31% of the total published authors, indicating that a core author group had not been formed. The author co-occurrence network map shows that 12 major collaborative teams centered on Hagberg Henrik, Wang Xiaoyang, Dammann Olaf, etc have been formed, covering 114 authors, generating 720 collaborations, and the total linkage intensity reaches 870, as shown in Figure 6. However, a considerable number of authors have not been integrated into these main cooperation models.

**Table 3:** The top 10 authors in terms of the number of published articles.

Author	Volume of publications	Total link strength
Romero Roberto	10	78
Sebire Guillaume	9	32
Hagberg Henrik	7	59
Burd Irina	7	25
Wang Xiaoyang	6	67
Gressens Pierre	6	51
Mallard Carina	6	40
Dammann Olaf	6	20
Zhang Xiaoli	5	59
Jantzie Lauren L	5	27

### 3.5. Co-occurrence analysis of keywords

**Figure 7.**
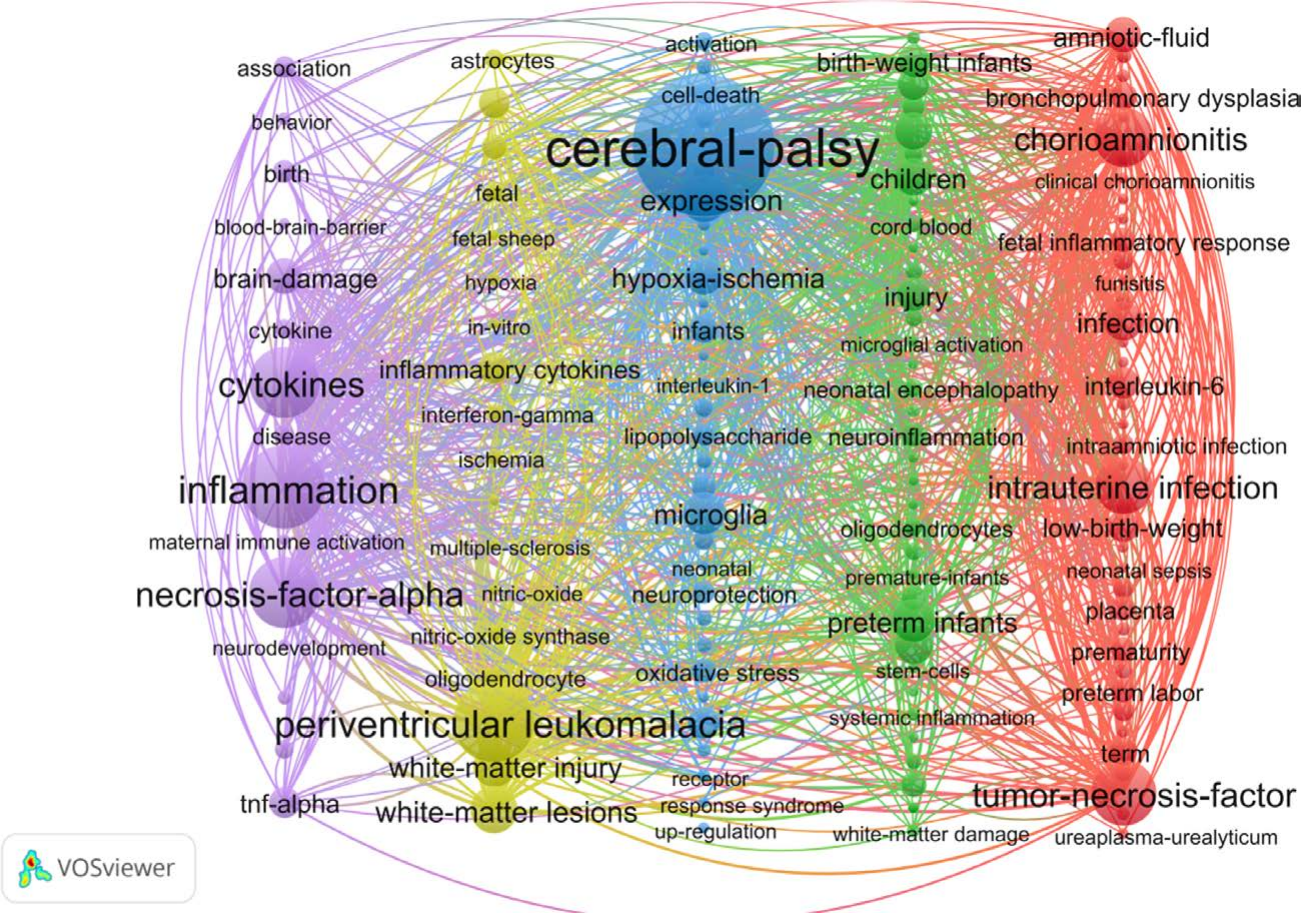
Keyword co-occurrence map. The node size is positively correlated with the frequency of keywords. The connection lines represent the co-occurrence frequency, and each column represents a cluster.

A total of 1527 keywords are involved. Excluding the keywords with the same research topic, the top ten keywords in terms of frequency are shown in Table 4. Visual analysis was conducted on keywords with a frequency of ≥5. The keyword co-occurrence map shows that 140 keywords were identified and divided into 5 different clusters. The frequency of keyword occurrence determines the size of the node, and the thickness of the connection between nodes reflects the co-occurrence frequency of the 2 keywords. The thicker the connection, the higher the co-occurrence frequency, that is, the closer the connection between the 2 keywords in the research content. See Figure 7.

**Table 4:** The top 10 keywords ranked by frequency.

Keywords	Frequency	Total link strength
Inflammation	70	489
Periventricular leukomalacia	68	531
Necrosis factor-alpha	57	424
Tumor necrosis factor	51	388
Chorioamnionitis	45	346
Intrauterine infection	43	339
Expression	34	230
White matter lesions	33	250
Preterm infants	30	212
Microglia	29	182

### 3.6. Keyword clustering analysis

**Figure 8.**
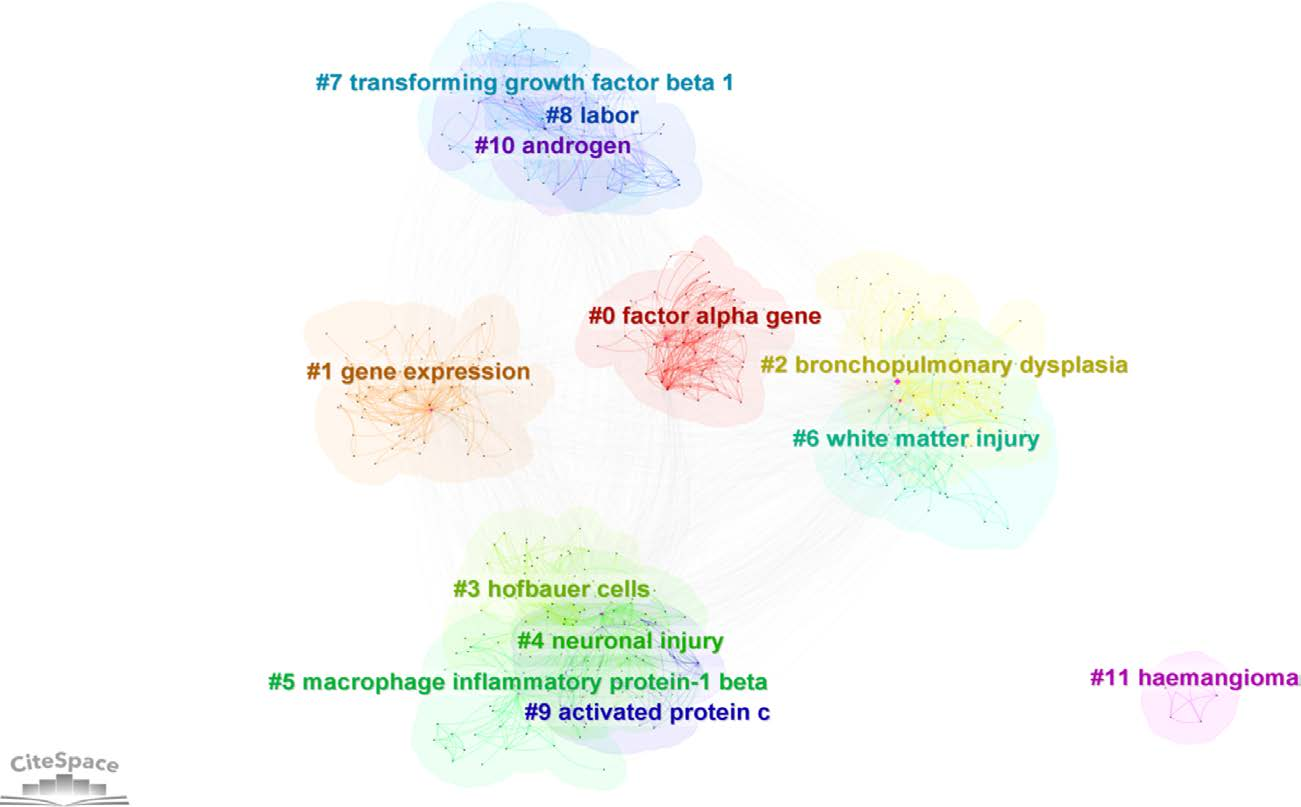
Keyword clustering graph. The tags are high-frequency keywords in the clustering, reflecting the research focus in this field. The intersection of the clustering plates indicates that there is an inherent correlation among the research topics.

A total of 12 clustering labels were obtained, as shown in Figure 8. It can be known from the graph information window that *Q* = 0.428 (>0.3) and *S* = 0.754 (>0.7), indicating that the credibility of this clustering is relatively high. The key points of research in this field can be divided into 4 categories: Inflammation and immune response (#0, #3, #5, #7, and #9), perinatal injury, and hypoxia–ischemia (#2, #4, #6, and #8), genetics and molecular regulation (#1 and #10), and other potential associated factors (#11).

### 3.7. Keyword emergence analysis

**Figure 9.**
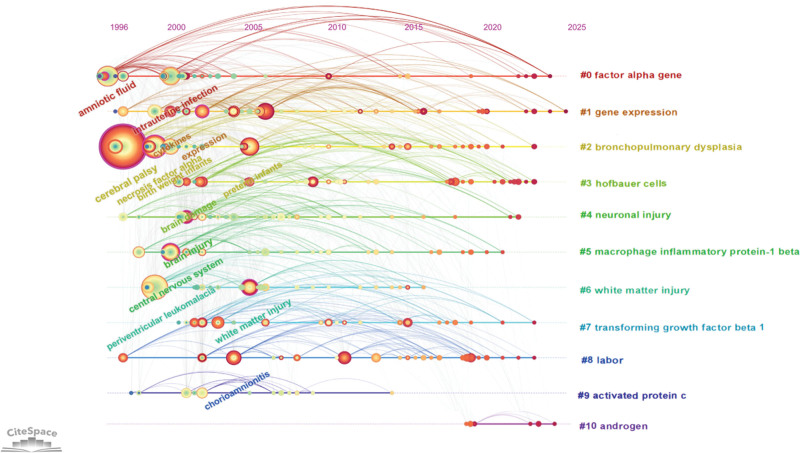
Keyword clustering timeline map. Display the first appearance and duration of each clustered keyword.

**Figure 10.**
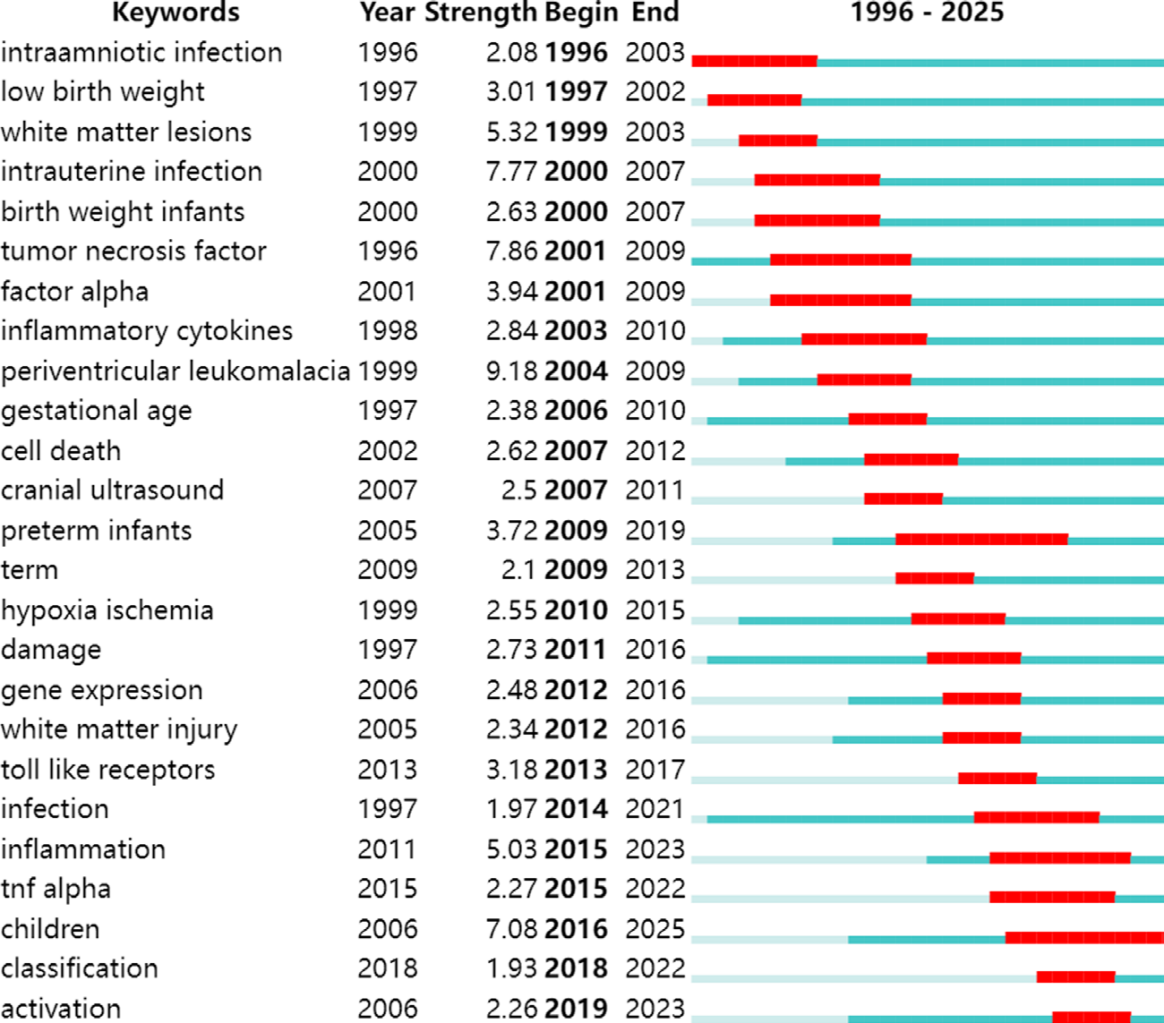
Keyword emergence graph. The blue line represents the time axis, and the red line segments indicate the emergent duration.

The timeline graph of keyword clustering shows that the significant change in keyword frequency over time indicates a change in the research focus, as shown in Figure 9. The minimum burst duration was set to 5 years, and 25 emergent words were obtained, as shown in Figure 10. The one with the greatest emergence intensity was periventricular leukomalacia, and the one with the longest emergence time was preterm infants. Overall, the study focused on basic factors such as early infection and birth weight. Gradually delve into the research on mechanisms such as cytokines and brain injury-related lesions. In recent years, research related to inflammation and the child population has become a new hotspot, reflecting the continuous expansion and deepening of the research on the pathogenesis of cerebral palsy.

## 4. Discussion

### 4.1. Research status quo

With the continuous advancement of medical research technology, the pathogenesis of CP has gradually attracted widespread attention, and the role of cytokines in it has begun to be noticed. From 1996 to 2025, this field as a whole showed fluctuations but no significant growth trend. This phenomenon may be related to the complexity of basic research on CP and the bottleneck of clinical transformation. Although the core role of cytokines as inflammatory mediators has gradually been confirmed, there are many repetitive studies on the mechanism of a single factor, and the emergence of breakthrough theories or technologies (such as multi-factor combined intervention) has not yet formed a scale effect. From the perspective of cooperation, the high subdivision of research directions in the field makes the research content of different teams vary greatly, and it is difficult to find points of interest in joint research, resulting in few opportunities for cooperation. At the same time, different regions have different levels of economic development, uneven distribution of scientific research resources, and the lack of advanced equipment and research funds in some regions, limiting the possibility of cooperation. Therefore, researchers should be provided with opportunities to display research results and exchange research experiences by building platforms, holding academic conferences, and sharing data, so as to improve research efficiency and level.

### 4.2. Analysis of the core research areas

Through keyword co-occurrence and cluster analysis, it was clearly found that inflammation and immune response have become the core research topics in the literature related to the pathogenesis of CP. In the co-occurrence network, tumor necrosis factor-α (TNF-α), macrophage inflammatory protein-1β, transforming growth factor-β1, Hofbauer cells, and activated protein C play an important role, highlighting their recognized role in CP-related inflammatory processes.^[8,9]^ These molecules are often placed in the context of the study of immune activation pathways, and an imbalance between pro-inflammatory and anti-inflammatory responses may be a potential driver of nerve damage.^[10–13]^ Among them, TNF-α occupies a central position. Inflammatory cytokines such as TNF-α dominate in the blood and cerebrospinal fluid of children with CP. A large number of nerve cells undergo apoptosis or necrosis, weakening the neuroprotective effect. A similar “waterfall” cascade immune-inflammatory response occurs, resulting in more severe brain injury and ultimately leading to the occurrence and development of CP.^[14–16]^ Therefore, the significant differences and important roles of TNF-α under normal physiological and pathological conditions make it a key object for studying the relationship between cytokines and CP.

Similarly, perinatal injury, and hypoxia–ischemia are another important research area for CP. In the co-occurrence network, terms such as periventricular leukomalacia, bronchopulmonary dysplasia, and neuronal injury are not only highly co-occurring, but also highly related to themes. These keywords often appear together with cytokine-related terms, which supports the idea that “cytokine-mediated mechanisms link perinatal adverse events to white matter damage and abnormal neurodevelopment.”^[17–19]^ The bibliometric structure shows that research in this field is often cross-integrated with clinical observations related to preterm infants and low birth weight infants, further highlighting the translational medical value of these pathways.

Furthermore, tags such as gene expression and androgen indicate that genetic factors may affect an individual’s susceptibility to CP by regulating cytokine signaling pathways. Finally, although the occurrence of hemangiomas did not form significant clusters, its potential association with abnormal vascular development deserves attention, which may involve the impact of abnormal cerebral blood flow on the cytokine microenvironment.

### 4.3. Research frontiers and trend evolution

Early CP studies mainly focused on perinatal high-risk factors. However, simple clinical observation cannot fully explain the pathogenesis of CP. Therefore, research has gradually shifted to the molecular level to explore the underlying pathophysiological processes. After entering the 21st century, the emergence of cytokines, TNF-α and periventricular white matter softening, etc, marked that CP research entered the stage of mechanism exploration. Periventricular leukomalacia, as a typical manifestation of brain injury in premature infants, is closely related to the motor dysfunction of CP. Its occurrence mechanism involves ischemia-reperfusion injury, microglial activation and the release of pro-inflammatory cytokines. Inflammatory factors such as TNF-α, IL-1β, and IL-6 can exacerbate white matter injury through pathways such as blood–brain barrier disruption, oligodendrocyte apoptosis, and myelin formation disorders, ultimately leading to motor and cognitive dysfunction.^[6,20–22]^ In recent years, the rise of keywords such as “inflammation” and “children” reflects the expansion of CP research towards inflammation-mediated neurodevelopmental disorders and long-term functional prognosis. More and more evidence indicates that CP is not merely a static brain injury, but a dynamic development process involving persistent neuroinflammation and abnormal synaptic plasticity. The long-term activation of microglia, the imbalance of pro-inflammatory and anti-inflammatory cytokines, and the dysregulation of glial cell–neuron interactions may play a key role in the progressive motor and cognitive disorders of children with CP.^[23,24]^ It is notable that “preterm infants” is the keyword with the longest emergence time, highlighting the core position of premature infants in CP research. With the advancement of neonatal intensive care technology, the survival rate of extremely premature infants (<28 weeks) has significantly increased, but the incidence of neurodevelopmental disorders such as CP has not decreased simultaneously, suggesting that a simple increase in survival rate has not completely solved the problem of neuroprotection. The mechanism of brain injury in premature infants is complex, involving the vulnerability of the immature blood–brain barrier, the systemic inflammatory response after intrauterine infection, and oxidative stress after hypoxia and ischemia, etc. Therefore, neuroprotection strategies for premature infants, such as prenatal anti-inflammatory treatment, stem cell therapy and early rehabilitation intervention, have become important research directions at present.

### 4.4. Future research directions and clinical transformation

Through bibliometric analysis, although important progress has been made in the study of the molecular mechanism of CP, there are still many challenges. On this basis, efforts should be strengthened in the following aspects in the future: first, with the help of systems biology and multi-omics integrated analysis, a cytokine regulatory network model should be constructed to analyze the pathogenesis of CP from a higher dimension; The second is to promote multicenter and large-sample clinical research, combined with longitudinal follow-up data, to establish an association model between cytokine profiles and CP subtypes. The third is to explore new strategies for targeted intervention to improve the accuracy and safety of treatment.

### 4.5. Limitations

This study only retrieved WOS, which might have omitted valuable literature from other databases, resulting in the research results being unable to comprehensively reflect the global research situation and affecting the accurate grasp of the overall picture of this field. Although some research hotspots and trends have been clarified, the research on the specific mechanism of cytokine action is not in-depth enough, and its dynamic changes and regulatory mechanisms in the complex pathological process of CP are still unclear. Furthermore, although the screening of keywords and cluster analysis are based on objective data and algorithms, they may still be affected by subjective factors and have a certain impact on the final research results. Therefore, in the future, the scope of data collection should be broadened, multisource literature should be integrated, and multidisciplinary methods should be combined to conduct in-depth research on the microregulatory mechanism of cytokines, reveal the core of the disease, and deepen the understanding of this field. Meanwhile, combined with other research methods, such as content analysis method and expert interviews, the results of visual analysis are verified and supplemented.

## 5. Conclusion

This study, through visual analysis, systematically has integrated the global research context of cytokines and the pathogenesis of CP for the first time. It was found that inflammation regulation, perinatal immune activation and neural repair are the core research chains, and international cooperation and interdisciplinary integration are the keys to breaking through the existing bottlenecks. In conclusion, cytokine research provides a key entry point for revealing the pathogenesis of CP, but its complex regulatory network and clinical transformation challenges still require collaborative innovation from global scientific research teams. With the innovation of technical means and the in-depth integration of multiple disciplines, the precise prevention and treatment strategy targeting cytokines is expected to become a new paradigm for the diagnosis and treatment of CP.

## Author contributions

**Formal analysis:** Yan Yang.

**Supervision:** Yan Yang.

**Writing – original draft:** Zhiliang Cao.

**Writing – review & editing:** Zhiliang Cao.
